# Molecular Detection of Gastrointestinal Pathogens Using a Multiplex Gastrointestinal Panel and Their Associations With Demographic and Clinical Characteristics

**DOI:** 10.7759/cureus.115820

**Published:** 2026-09-05

**Authors:** Diego Villalobos-León, Mauricio Castillo-Salazar, Valentina Flores-Gallardo, María Fernanda Villavicencio-Pérez, Fátima Mariamne Gómez-Navarro, Carlos Andrey Diosdado-Franco, Luis Gerardo Balcázar-Ochoa, Laura Gómez-Virgilio

**Affiliations:** 1 Clinical and Pharmaceutical Sciences, Justo Sierra University, Mexico City, MEX; 2 Laboratory and Blood Bank, Hospital Angeles Lindavista, Mexico City, MEX; 3 Pharmacy, Universidad Autónoma del Estado de Morelos, Cuernavaca, Morelos, MEX; 4 Medicine, Hospital Angeles Lindavista, Mexico City, MEX; 5 General Management, Hospital Angeles Arboledas, Tlalnepantla de Baz, MEX; 6 Physiology, School of Medicine, Universidad Nacional Autónoma de México, Mexico City, MEX; 7 Research and Teaching Coordination Office, Hospital Angeles Lindavista, Mexico City, MEX

**Keywords:** acute gastroenteritis, antimicrobial stewardship, e. coli pathotypes, gastrointestinal pathogens, molecular diagnostics, seasonal trends

## Abstract

Background

Acute gastroenteritis causes morbidity across all age groups, and timely, effective identification of its etiologic agents is essential for clinical management and antimicrobial stewardship. Conventional diagnostic methods have limited sensitivity and often fail to detect coinfections or specific bacterial pathotypes. In contrast, molecular methods, such as FilmArray panels, are rapid, highly sensitive, and capable of detecting coinfections. This study aimed to characterize gastrointestinal pathogens detected by the BioFire FilmArray Gastrointestinal Panel (BioFire Diagnostics, LLC, Salt Lake City, UT, USA; a bioMérieux company) and to evaluate their association with demographic and clinical characteristics among patients at Hospital Angeles Lindavista, Mexico City, Mexico.

Methods

A retrospective observational study conducted from January 2022 to July 2025 included all patients who underwent gastrointestinal molecular testing. Demographic data, clinical characteristics, and multiplex PCR results were extracted from electronic medical records and the FilmArray database. Data were analyzed using descriptive statistics, logistic regression, Fisher’s exact test, and Spearman correlation.

Results

An analysis of 1,120 samples found an overall positivity rate of 906 (81%). Coinfections were present in 543 (49%) cases. The most common pathogens were enteropathogenic *Escherichia coli *(*E. coli, *EPEC; 374, 33.4%); enteroaggregative *E. coli *(EAEC; 281, 25.1%); and enterotoxigenic *E. coli* (ETEC; 231, 20.6%). Rotavirus A (206, 18.4%) and norovirus GI/GII (168, 15%) were the most frequent. Positivity was highest in children under five (147, 84.48%) and lowest in adults over 60 (129, 73.71%). Seasonal patterns showed unexpected peaks of rotavirus in spring-summer and of *Cyclospora cayetanensis* in summer. No significant association was found between pathogen presence and comorbidities, likely due to many missing clinical details. A negative correlation among major *E. coli* pathotypes suggests competitive exclusion.

Conclusion

The BioFire FilmArray Gastrointestinal Panel identified pathogen patterns that conventional methods miss. Age and seasonal trends highlight the need for localized epidemiological surveillance. These results encourage the use of molecular diagnostics in routine assessments of gastrointestinal infections and support antimicrobial stewardship efforts. Future studies should include clinical severity metrics and evaluate patient outcomes to connect molecular detection with clinical significance.

## Introduction

Gastrointestinal infections are a common reason for seeking medical care and a global public health problem. While pediatric patients are known to be the most vulnerable, these infections also represent a significant cause of morbidity and mortality in adults, particularly those with comorbidities [1,2]. Rapid, timely diagnosis improves treatment, disease course, and prognosis and supports epidemiological surveillance. One of the greatest challenges in establishing a diagnosis is the wide range of pathogens that can cause infection, including viruses, bacteria, and parasites, acting alone or in combination [3].

Current diagnostic testing options include culture, staining, and antigen detection. These methods may require collecting multiple samples, involve extensive sample preparation, and have low sensitivity and prolonged turnaround times. Traditionally, the gold standard for microbiological diagnosis includes culture for *Salmonella *spp., *Shigella *spp., *Campylobacter*, and *Escherichia coli* (*E. coli*), as well as rapid tests for rotavirus. However, these agents account for only 25%-30% of diarrheal syndromes [4]. Molecular detection tests have expanded the capacity to detect pathogens. However, their availability is limited for most of the population due to their high cost and limited access in public health institutions [5]. For the FilmArray Gastrointestinal Panel, the Food and Drug Administration (FDA) has validated its clinical use, allowing detection in a single reaction of 22 enteric pathogens (13 bacteria, five viruses, and four parasites) and resistance genes [6].

In Mexico, this technique is not widely available in public health services and is used only in specific cases or in the private sector [7]. Therefore, describing its use in the general population is relevant, as is its association with clinical characteristics such as diagnosis, comorbidities, and risk factors. This study aims to identify and describe the microorganisms detected in patients who underwent a gastrointestinal molecular panel at Hospital Ángeles Lindavista, Mexico City, Mexico, from January 2022 to July 2025, without establishing a definitive cause-and-effect relationship, and to evaluate their correlation with demographic and clinical features. To this end, we will conduct a retrospective, descriptive analysis, with the results intended to inform improvements in patient care, diagnosis, treatment, antimicrobial stewardship decisions, and epidemiological surveillance.

## Materials and methods

Study design

We conducted a retrospective, observational, cross-sectional study at Hospital Angeles Lindavista from January 2022 to July 2025. Its goal was to identify and examine gastrointestinal pathogens, including bacteria, viruses, and parasites, detected by the BioFire FilmArray Gastrointestinal Panel (BioFire Diagnostics, LLC, Salt Lake City, UT, USA; a bioMérieux company). The study aimed not only to understand epidemiological patterns but also to associate these pathogens with patients' demographic and clinical features; it included all patients who received a gastrointestinal molecular panel, whether as outpatients or while hospitalized. Analyzed variables included demographics, patient type, comorbidities, risk factors, diagnoses, detected pathogens, and infection type. Data sources included the FilmArray multiplex PCR system database (by bioMérieux) and the Hospital Angeles Lindavista Electronic Clinical History (ECH) database.

Laboratory testing

Samples examined were stool specimens. We identified gastrointestinal pathogens using the BioFire FilmArray Gastrointestinal Panel multiplex PCR system. This certified device combines nucleic acid extraction, reverse transcription, first-stage multiplex PCR, and second-stage nested PCR within a single disposable pouch. It employs freeze-dried reagents and multiple primers optimized for a common annealing temperature, allowing simultaneous qualitative detection of 22 gastrointestinal pathogens, such as diarrheagenic *E. coli *types, *Campylobacter*, *Salmonella*, *Vibrio*, *Yersinia enterocolitica*, *Clostridioides* *difficile* (*C. difficile*) toxin A/B, *Cryptosporidium*, *Cyclospora*, *Entamoeba histolytica*, *Giardia lamblia*, adenovirus F40/41, astrovirus, norovirus GI/GII, rotavirus A, and sapovirus. All steps occur inside sealed blister compartments in the pouch, reducing manual handling and contamination risk and delivering results in about an hour. We performed tests following the manufacturer's protocols. Results labeled "detected" or "not detected" were considered valid. If a process failed, we marked the result "invalid" and repeated the test. This approach improves diagnostic speed and helps detect coinfections but has limitations: sensitivity varies among targets; it does not quantify microbial load, so it cannot differentiate active infection from colonization or residual nucleic acids, and it detects only organisms within the panel and cannot identify pathogens outside it. A negative result does not rule out microorganisms not among the panel's targets. Therefore, we interpreted results alongside clinical presentation and additional diagnostics.

Data source

We compiled a retrospective database from Hospital Angeles Lindavista for the specified period, including data from 1,120 patients. We de-identified the patients' data and standardized and grouped variable coding for analysis.

Statistical analysis

Statistical analyses were conducted using R version 4.5.1 (R Foundation for Statistical Computing, Vienna, Austria) in RStudio (Posit Software, PBC, Boston, MA, USA).

Descriptive Analysis

Categorical variables were summarized with their absolute counts and percentages. For the continuous variable (age), we assessed the data distribution and reported the mean and standard deviation (SD). Overall and age-specific positivity rates were calculated by dividing detected cases by the total samples processed.

Seasonality Analysis

We examined the monthly detection frequency of the top 10 most common pathogens over the study period (2022-2025) to identify seasonal trends and periodic fluctuations.

Inferential Analysis and Associations

To analyze links between clinical variables (such as comorbidities and risk factors) and pathogen presence, we used binary logistic regression to calculate odds ratios (ORs) with 95% confidence intervals (CIs). For small subgroups (n < 5), we used Fisher's exact test for sensitivity checks.

Finally, to investigate microbial interactions (coinfections versus mutual exclusion), we created a correlation matrix based on Spearman's rank correlation coefficient, considering only statistically significant interactions.

All statistical tests used a significance threshold of p < 0.05 (two-tailed).

Ethical considerations

The Research Ethics Committee of Hospital Angeles Pedregal approved the study protocol (approval number: HAP 2786). All procedures followed the ethical standards outlined in the Declaration of Helsinki. The committee granted a waiver of informed consent for this retrospective study because of its minimal-risk design. Confidentiality and data protection were maintained throughout the study.

## Results

Characteristics of the study population

A total of 1,120 samples from patients with suspected gastrointestinal infection treated at Hospital Angeles Lindavista between January 2022 and July 2025 were analyzed. The mean age of the patients was 33.6 years (SD ± 23.1). A balanced gender distribution was observed, with 616 (55%) female patients. Most samples (1,028, 92%) were from hospitalized patients, while 92 (8%) were from outpatients (Table 1).

**Table 1:** Clinical and demographic characteristics of the study population. For the age variable, the values correspond to the mean and standard deviation (SD). For the other values, these correspond to the number of cases (n) and the percentage of the total population (%).

Characteristic	N = 1,120
Age (years)	34 (23)
Age group	
Children (0–5 years)	174 (16%)
Children (6–17 years)	152 (14%)
Adults (18–59 years)	619 (55%)
Older adults (>60 years)	175 (16%)
Gender	
Female	616 (55%)
Male	504 (45%)
Patient type	
Outpatient	92 (8.2%)
Hospitalized	1,028 (92%)

Regarding the diagnosis, 878 (79%) patients were admitted with acute gastroenteritis, 74 (6.6%) with gastroenteritis secondary to *C. difficile*, and 28 (2.5%) had no reported diagnosis. In 761 (68%) and 709 (64%) of the analyzed population, the presence of comorbidities and risk factors was unknown, respectively. Among patients with available information, hypertension (48, 4.3%) and diabetes (41, 3.7%) were the most frequent comorbidities, while immunosuppression (27, 2.4%) and the presence of concomitant infections (15, 1.3%) were among the most common risk factors (Table 2).

**Table 2:** Admission diagnoses, comorbidities, and risk factors among patients. Each pair of data points corresponds to the number of cases (n) and the percentage of the total population (%).

Characteristic	N = 1,120
Admission diagnosis	
Acute gastroenteritis	878 (79%)
Gastroenteritis secondary to *Clostridioides difficile*	74 (6.6%)
Other (postoperative, oncologic, neurologic, etc.)	49 (4.4%)
No diagnosis	28 (2.5%)
Abdominal and/or urinary sepsis	22 (2.0%)
Urinary tract infection	21 (1.9%)
Respiratory tract infection	13 (1.2%)
Unspecified gastropathy	12 (1.1%)
Unknown	9 (0.8%)
Infectious colitis	7 (0.6%)
Inflammatory bowel disease/Crohn’s disease -ulcerative colitis	4 (0.4%)
Comorbidities	
Unknown	761 (68%)
None	191 (17%)
Hypertension	48 (4.3%)
Diabetes	41 (3.7%)
Cancer	21 (1.9%)
Chronic kidney disease	18 (1.6%)
Other (neurologic, respiratory, rheumatologic, etc.)	13 (1.2%)
Hypothyroidism	11 (1.0%)
Heart failure	9 (0.8%)
Unspecified gastropathy	4 (0.4%)
Liver failure	3 (0.3%)
Risk factors	
Unknown	709 (64%)
None	281 (25%)
Other (diabetes, hypertension, Parkinson’s disease)	28 (2.5%)
Immunosuppression	27 (2.4%)
Concomitant infection	15 (1.3%)
Poor diet	11 (1.0%)
Short breastfeeding duration	11 (1.0%)
Post-surgical	10 (0.9%)
Renal failure	10 (0.9%)
Hypothyroidism	5 (0.4%)
Irritable bowel syndrome	5 (0.4%)
HIV	4 (0.4%)

Prevalence and profile of gastrointestinal pathogens

The overall positivity rate for the panel was 906 (81%). Among positive cases, coinfections were common: 363 (32%) had single infections (one pathogen), while 543 (49%) had multiple infections (two, three, or more than four pathogens detected simultaneously) (Table 3).

**Table 3:** Gastrointestinal panel results and infection burden in the study population. Each pair of data points corresponds to the number of cases (n) and the percentage of the total population (%).

Characteristic	N = 1,120
Gastrointestinal panel result	
Detected (positive)	906 (81%)
Not detected (negative)	214 (19%)
Classification by number of pathogens	
No infection	214 (19%)
Single infection (1 pathogen)	363 (32%)
Double infection (2 pathogens)	258 (23%)
Triple infection (3 pathogens)	166 (15%)
Multiple infections (>4 pathogens)	119 (11%)

Pathogenic bacteria dominated the microbiological profile. The most frequently detected microorganisms were enteropathogenic *E. coli* (EPEC), 374 (33.4%), followed by enteroaggregative *E. coli* (EAEC), 281 (25.1%), and enterotoxigenic *E. coli* (ETEC), 231 (20.6%). Among viral agents, rotavirus A was most prevalent (206, 18.4%), followed by norovirus GI/GII (168, 15%) (Figure 1).

**Figure 1:**
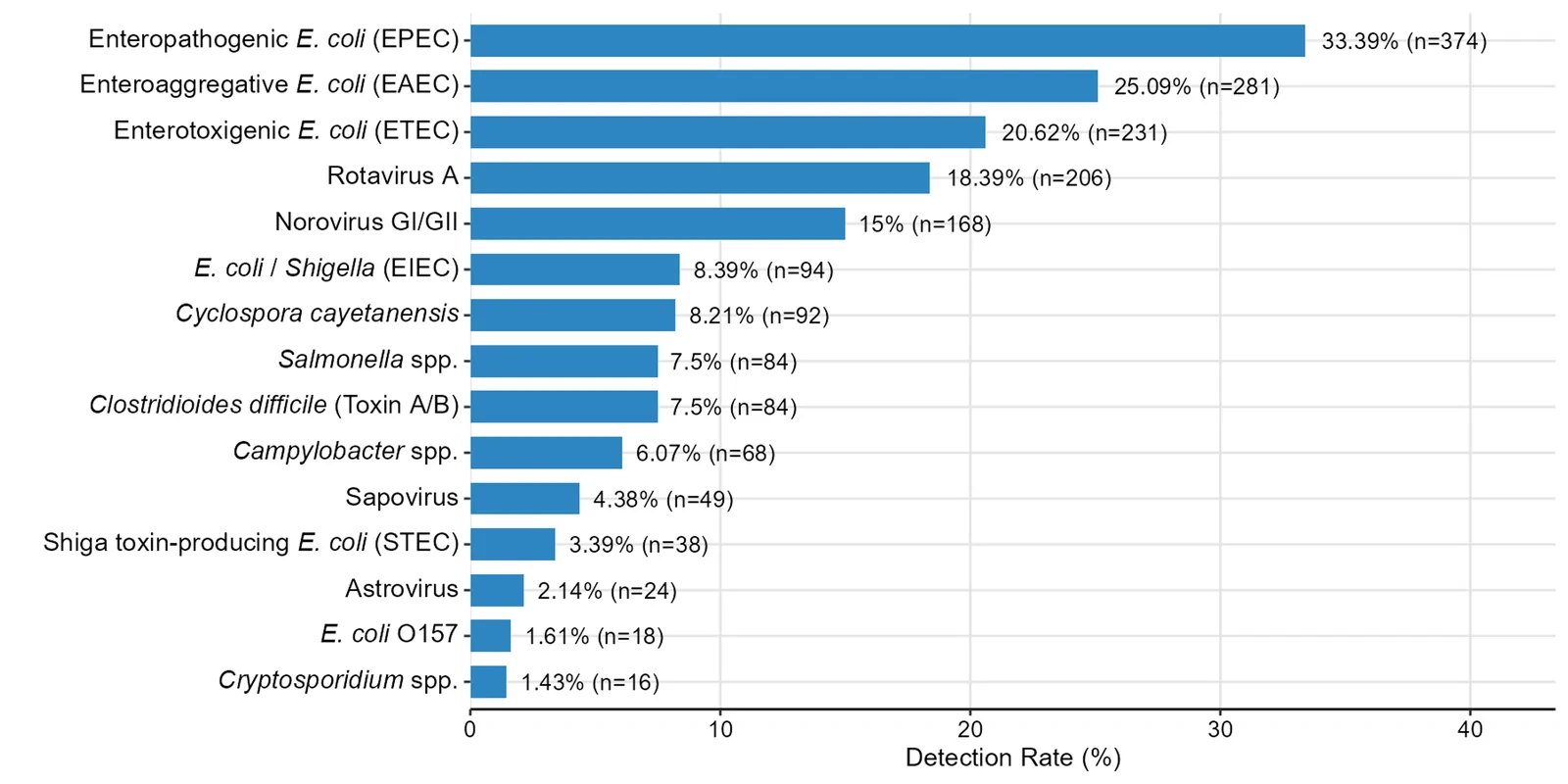
Frequency of enteric pathogens identified through a multiplex gastrointestinal panel. Each horizontal bar represents a pathogen, showing detection rates and counts. The data primarily indicate that *Escherichia coli* (*E. coli*) pathotypes dominate. Viral pathogens such as rotavirus A and norovirus GI/GII are also notably present. Less common are *E. coli* O157 at 18(1.61%) and *Cryptosporidium* spp. at 16(1.43%). Overall, the figure illustrates pathogen burden and relative frequencies within the study group.

By age group, the molecular panel showed high detection rates (>73%) across all groups (Figure 2). Nevertheless, positivity declined with age: children aged 0 to five years had the highest rate at 147/174 (84.48%), closely followed by the six-to 17-year group at 128/152 (84.21%). Conversely, adults over 60 years had the lowest detection rate at 129/175 (73.71%), suggesting a potentially higher prevalence of non-infectious or undetectable causes in this age group.

**Figure 2:**
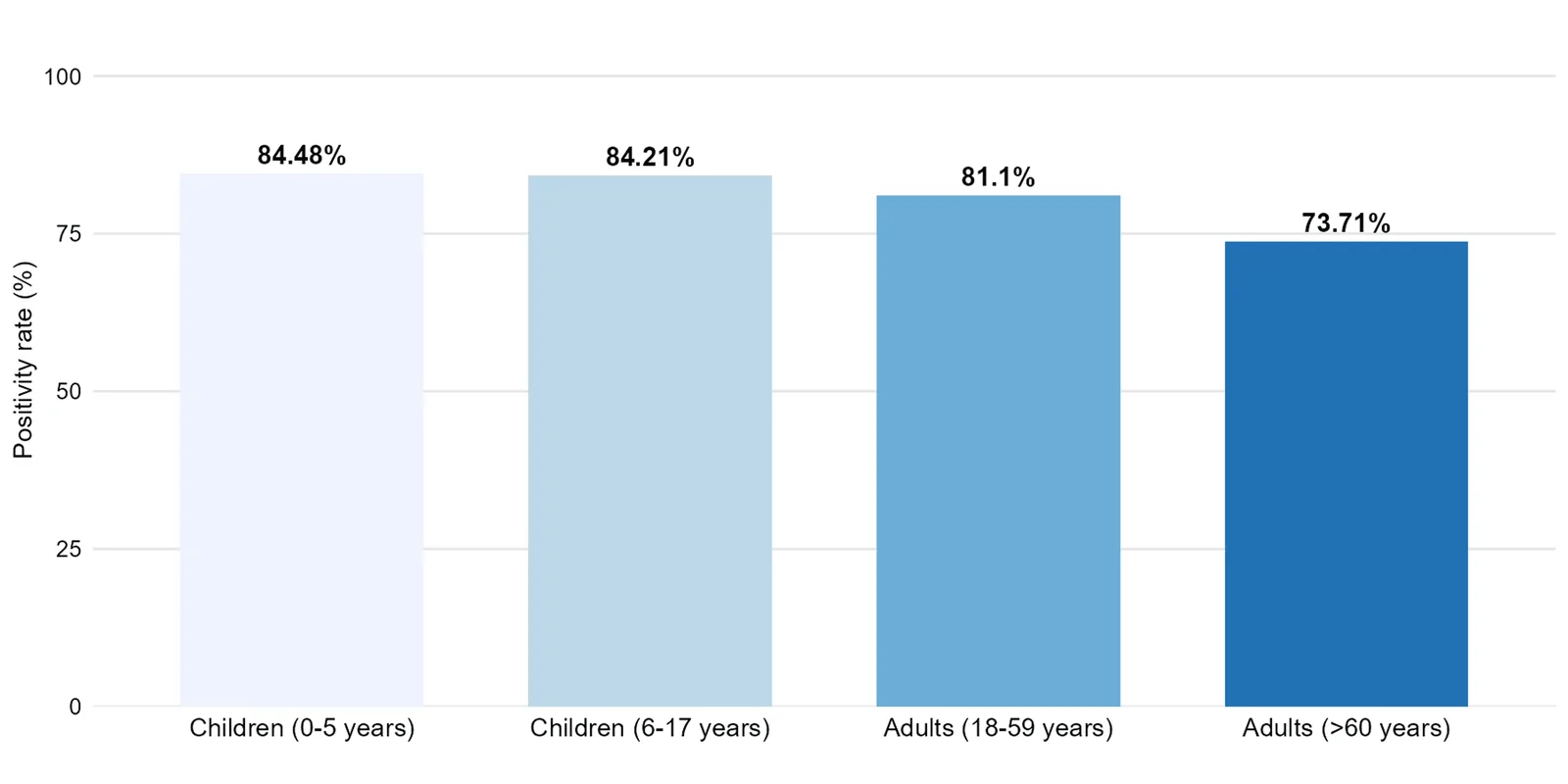
Gastrointestinal panel positivity rate stratified by age group. The highest positivity rates are observed in children aged 0 to five years. Adults aged 18 to 59 show a slightly lower rate of 502/619 (81.1%), while those over 60 have the lowest rate at 129/175 (73.71%). These data suggest that positivity decreases with age, highlighting age-related differences in detecting the pathogen.

Seasonality patterns

Monthly trend analysis revealed seasonal patterns (Figure 3). Unlike the common winter peaks seen elsewhere, rotavirus detections surged in spring and summer (March-June), aligning with peaks in bacterial pathogens such as *Salmonella* spp. and various *E. coli* strains. Furthermore, *C. difficile* cases peaked from May to July. *Cyclospora cayetanensis* also showed a distinct summer seasonal pattern.

**Figure 3:**
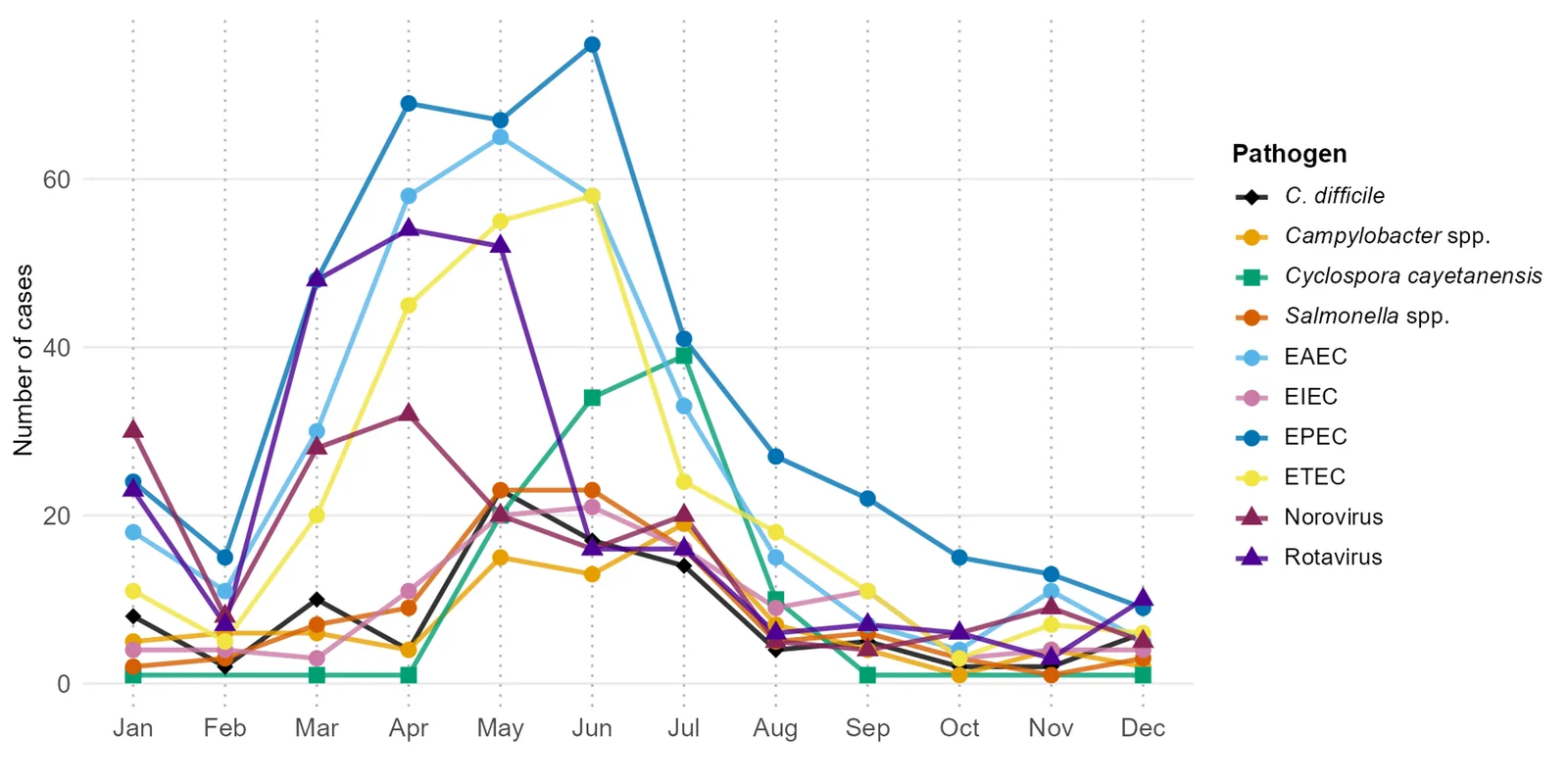
Monthly distribution of cases for multiple gastrointestinal pathogens. A seasonal pattern trend appears, with most pathogen cases increasing from April to July. The highest peaks are observed among *Escherichia coli* (*E. coli*) pathotypes, namely enteropathogenic *E. coli* (EPEC), enteroaggregative *E. coli* (EAEC), and enterotoxigenic *E. coli* (ETEC), with counts approaching 60 in late spring and early summer. Other pathogens such as *Campylobacter* spp., *Salmonella* spp., *Clostridioides difficile *(*C. difficile*), and enteroinvasive *E. coli* (EIEC) also exhibit a similar seasonal rise, though less pronounced. After July, case counts decline sharply and remain low for the rest of the year. Overall, the data highlight a distinct mid-year surge in enteric infections, consistent with typical seasonal epidemiological patterns.

Association with age group

Multivariate analysis showed a strong association between age and infection etiology (Figure 4). Compared with the adult group (18 to 59 years), pediatric patients (0 to five years) had a significantly higher probability of infection with viral agents, notably rotavirus (OR > 1, p < 0.05) and norovirus. This age group also had a higher probability of detecting bacteria such as *C. difficile* and *Campylobacter* spp. Other pathogens, such as EPEC and ETEC​​​​​​, were less likely to be isolated in children aged 0 to five years, and *Cyclospora cayetanensis* was less likely to be isolated in pediatric patients overall. Furthermore, *C. difficile* showed a trend towards higher detection in adults over 60 years of age, consistent with the hospital epidemiology of this pathogen.

**Figure 4:**
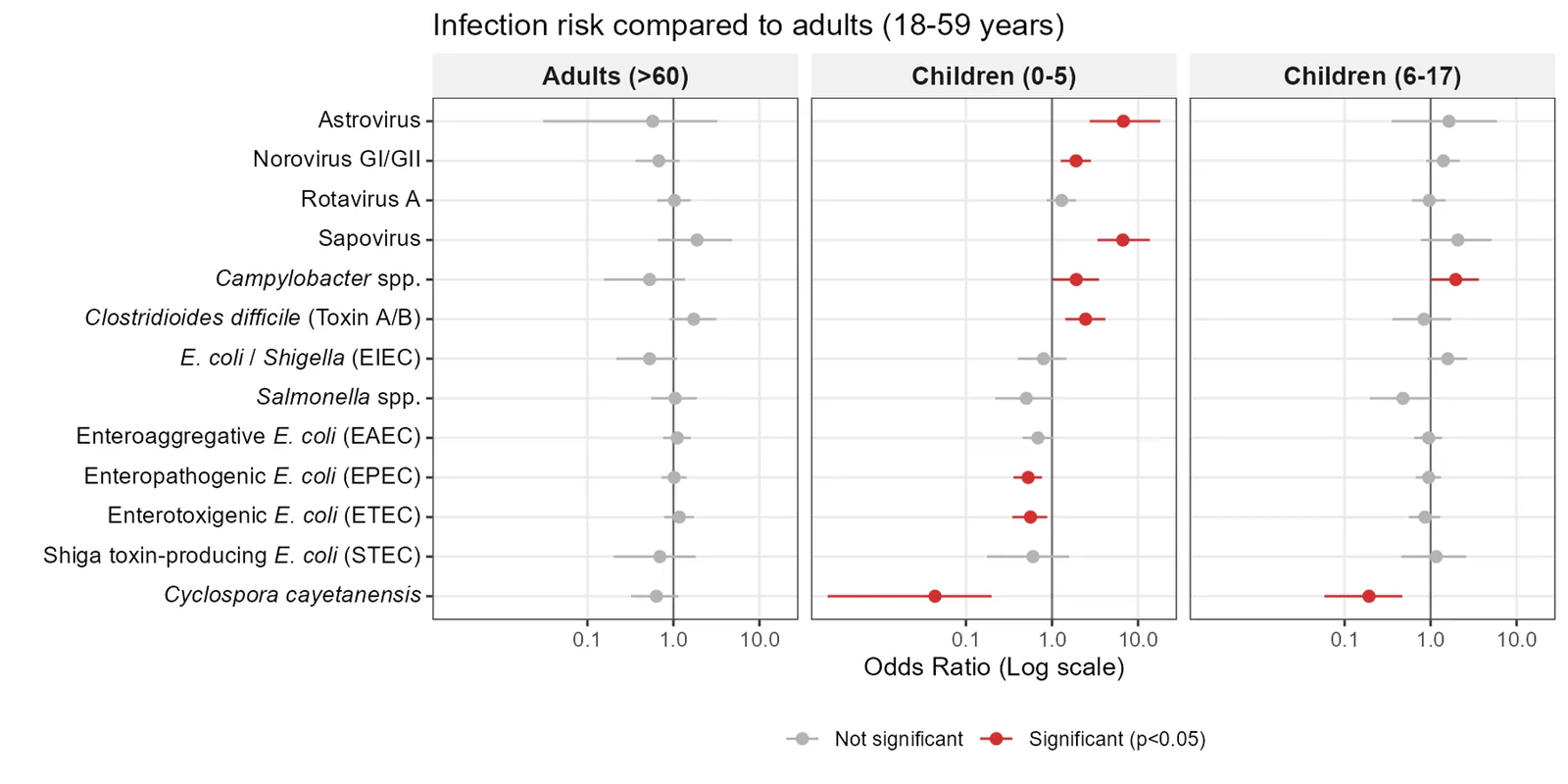
Comparative analysis of infection risk across age groups using odds ratios on a logarithmic scale. Three panels present the relative odds of pathogen detection among adults aged 60 and over, children aged 0 to five, and children aged six to 17, each compared with adults aged 18 to 59. The chart displays point estimates with CIs for different pathogens, using red markers to indicate statistically significant associations (p<0.05) and gray markers for non-significant results. It highlights age-related variations in susceptibility, showing which pathogens are more or less commonly detected in younger children and older adults compared with the adult reference group. *E. coli*: *Escherichia coli*

Comorbidities and risk factors

Sensitivity analyses examined how comorbidities (such as type 2 diabetes mellitus and systemic hypertension) (Figure 5) and risk factors (like immunosuppression) (Figure 6) influence susceptibility to specific pathogens. We excluded patients with missing data or those classified as 'unknown,' focusing only on those with confirmed diagnoses and healthy controls. Under this strict criterion, we found no significant associations for any condition (Fisher's exact test, p > 0.05), suggesting that acquiring these community pathogens occurs independently of metabolic comorbidities in this population.

**Figure 5:**
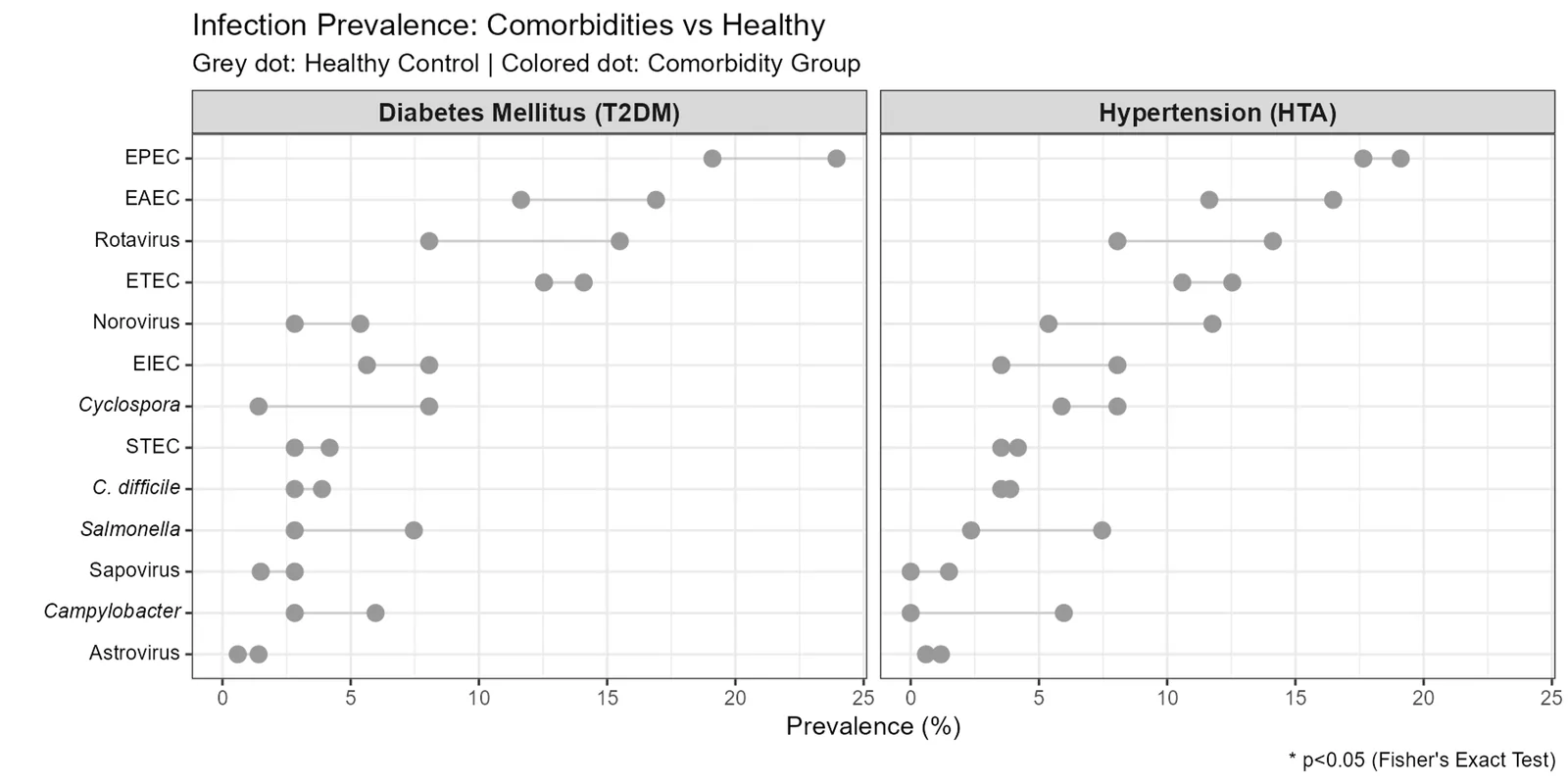
Comparison of the prevalence of multiple enteric pathogens between patients with comorbidities (type 2 diabetes mellitus (T2DM) or hypertension (HTA)) and healthy individuals. In the plot, each panel displays pathogen-specific prevalence, with gray markers indicating healthy individuals and colored markers representing participants with the relevant comorbidity. The x-axis shows prevalence (%) while the y-axis lists detected pathogens, including bacterial, viral, and protozoal agents. The two-panel layout allows for straightforward comparison of infection patterns across different comorbidity profiles. Statistically significant differences are indicated with an asterisk (p<0.05, Fisher’s exact test). Overall, the figure illustrates that no significant associations were found for any of the conditions.

**Figure 6:**
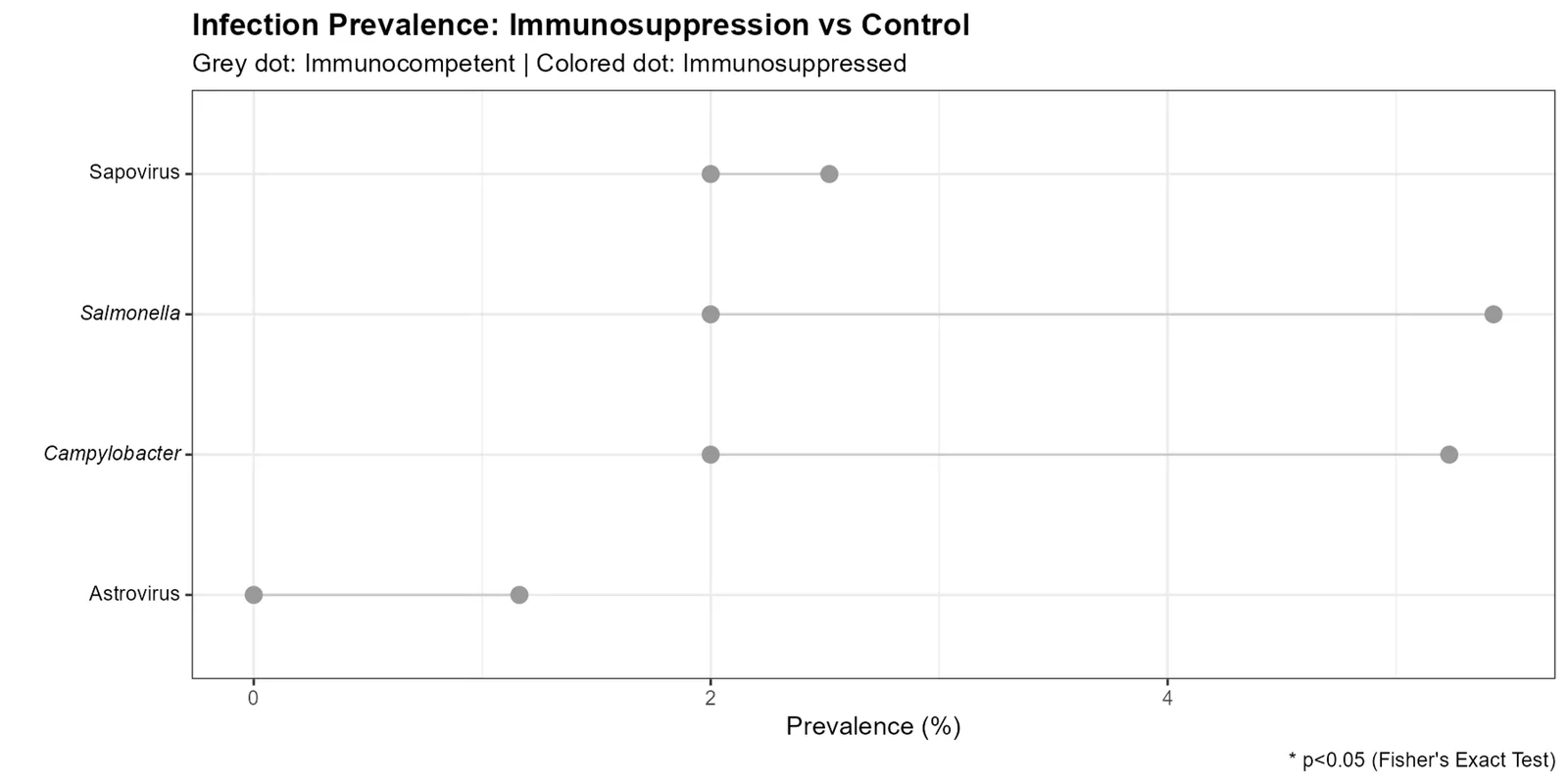
Comparison of the prevalence of enteric pathogens between immunosuppressed and immunocompetent patients. The scatter plot illustrates the prevalence of four enteric pathogens, namely, sapovirus, *Salmonella*, *Campylobacter*, and astrovirus, among immunocompetent (gray markers) and immunosuppressed (colored markers) individuals. Each data point indicates the proportion of infected individuals within each immune-status group. The x‑axis shows prevalence (%) while the y‑axis lists the pathogens, allowing for straightforward comparison of infection rates under different immune conditions. Statistically significant differences are indicated with an asterisk (p<0.05, Fisher’s exact test). Overall, the figure illustrates that no significant associations were found for any of the conditions.

Ecological interactions among pathogens

Spearman correlation analysis indicated predominantly negative interactions, or competitive exclusion, among the main *E. coli* pathotypes. Notably, EPEC detection was negatively correlated with both EAEC (r = -0.22, p < 0.001) and ETEC (r = -0.16, p < 0.05). We also observed a weak negative correlation between rotavirus and EPEC (r = -0.16), suggesting that coinfections involving these agents are less common than expected by chance.

## Discussion

Results from the microbiological profile of patients who underwent a gastrointestinal molecular panel at Hospital Ángeles Lindavista over 32 months showed an overall positivity rate of 906/1,1120 (81%), considerably higher than the 25%-30% observed with conventional methods. This finding aligns with studies by Buss et al. and Spina et al., reinforcing the clinical utility of multiplex molecular panels as a first-choice diagnostic tool for identifying the microorganism responsible for gastroenteritis in hospital settings [6,8].

On the other hand, the microbiological profile showed a clear predominance of *E. coli*, with EPEC the most frequent agent (374, 33.4%), followed by EAEC (281, 25.1%), and ETEC (231, 20.6%). Conventional methods do not differentiate among types of the same pathogen, such as *E. coli* in this case, leading to underdiagnosis of these strains. This underscores the importance of incorporating molecular technologies into the diagnostic algorithm for infectious gastroenteritis and applying them specifically in the hospital setting, which, in the studied population, accounted for 1,028 (92%) of the sample. We also observed a high rate of coinfection (543/11,120 (49%)), consistent with existing literature [9]. Clinically, the presence of multiple pathogens in the studied population highlights the need for highly sensitive, rapid, and precise detection of key microbiological agents, such as viruses, bacteria, and parasites. This approach enables timely identification of the etiological agents and supports better antimicrobial decision-making, emphasizing the importance of molecular testing to meet all therapeutic needs [6,10]. In contrast, Spearman's correlation revealed a negative relationship among *E. coli* pathotypes, including EPEC, EAEC, and ETEC, indicating that coinfections with these microorganisms are less frequent. Literature suggests that EPEC and ETEC both bind to the same intestinal receptor, glycoprotein 2, leading to direct competition for binding sites on the intestinal lining, where the presence of one can hinder the adhesion of the other [11].

The pediatric age group of 0 to five years had the highest positivity rate, at 147/174 (84.48%). This pattern may be attributed to several factors, mainly the integration of young children into group settings, especially daycare centers and preschools, where they have direct contact with other children. This facilitates the most common transmission mechanisms in this group, such as the fecal-oral route, as well as lapses in hygiene practices or handling contaminated shared objects [12]. Given this epidemiological context, greater susceptibility to acquiring microorganisms is observed, especially viral agents such as rotavirus A and norovirus GI/GII [13]. The declining trend in positivity rates with age is also noteworthy. This is evident in adults over 60 years of age, with a rate of 129/175 (73.71%), mostly attributable to non-infectious causes such as medication-related diarrhea, chronic comorbidities with persistent pro-inflammatory states, inflammatory bowel diseases, and microbiota dysbiosis due to advanced age [14,15]. However, among probable infectious causes in older adults, microorganisms with greater clinical and diagnostic complexity, such as *C. difficile*, were detected more frequently. This is associated with factors inherent to aging, such as ineffective immune responses and an altered intestinal environment that favor colonization by these infectious agents. Furthermore, geriatric patients are the age group with the longest hospital stays; therefore, prolonged antibiotic therapy regimens are used, which further alter the intestinal microbial environment and lead to various complications of gastrointestinal diseases [16].

The temporal pattern shows that microorganisms such as rotavirus, *Salmonella* spp., *Cyclospora cayetanensis*, and various *E. coli* subtypes predominate from March to June, coinciding with climatic changes in Mexico. Increased temperature and humidity favor their emergence and survival, facilitating transmission through water, contaminated food, and the fecal-oral route [17]. Additionally, cases of *C. difficile* were detected in May and July, which could be associated with other infections more common during the same warm-weather season, such as EPEC, enteroinvasive *E. coli*, and *Salmonella*, and consequently with increased antibiotic use [17,18].

The connection between a patient's chronic conditions and their susceptibility to gastrointestinal pathogens has been studied, with conditions like type 2 diabetes and systemic hypertension linked to a higher risk of infection. For example, diabetes can weaken immune responses and change gut microbiota composition, potentially encouraging microbial colonization [19]. Despite this, statistical analysis revealed no significant link between these conditions and the presence of specific pathogens (p > 0.05, Fisher's exact test). A key limitation is that 761 (68%) patients lacked recorded comorbidity data, and 709 (64%) lacked recorded risk factor information, substantially reducing the sample size and hindering detection of potential differences. Furthermore, because the study was conducted in private hospitals, where chronic illness management is often more effective, these results may not apply across clinical settings. Another limitation is the study design: as a retrospective, non-probabilistic study, it introduces potential selection and data-collection biases and limits causal inference. The fact that most participants were hospitalized also hampers applicability to community settings; key clinical severity measures such as symptom duration, treatment response, and length of hospital stay were not assessed. Moreover, the molecular panel detects pathogen presence but not load, which limits understanding of the significance of coinfections; in addition, molecular detection of a microorganism does not necessarily confirm it as the true causative agent, especially given colonization or asymptomatic presence. However, this test cannot detect microorganisms outside the commercial panel's predefined targets. Last, seasonal trend analyses limited to the most common pathogens may miss patterns involving less frequent agents.

Despite some limitations, the study provides a thorough characterization of gastrointestinal pathogens using the BioFire FilmArray Gastrointestinal Panel in a cohort of 1,120 patients. Multiplex molecular diagnostics have greatly enhanced pathogen detection, enabling identification of specific *E. coli* pathotypes and common coinfections that traditional methods often overlook. Furthermore, the study offers new epidemiological insights from a private hospital in Mexico, supporting antimicrobial stewardship and helping shape local diagnostic strategies.

Future research should use prospective designs, confirm clinical infection, incorporate clinical severity metrics, and assess patient outcomes to link molecular detection to clinical impact. Expanding similar analyses to other healthcare institutions could strengthen regional surveillance and support broader implementation of molecular diagnostic technologies across Mexico.

## Conclusions

The study found a high positivity rate, frequent coinfections, and variation in gastrointestinal pathogen circulation across demographic groups and seasons. These patterns underscore the importance of continuous regional surveillance to monitor shifts in pathogen activity.

Our findings indicate that molecular diagnostics provide clinically valuable information that enhances pathogen identification, may inform antimicrobial stewardship, and strengthens epidemiological monitoring. Despite limitations from missing clinical data and a retrospective design, this work offers relevant insights to improve diagnostic algorithms and to guide future prospective research correlating molecular detection with clinical outcomes.
